# Unusual enhancement of ~ 30 MeV proton flux in an ICME sheath region

**DOI:** 10.1186/s40623-021-01362-y

**Published:** 2021-01-29

**Authors:** Mitsuo Oka, Takahiro Obara, Nariaki V. Nitta, Seiji Yashiro, Daikou Shiota, Kiyoshi Ichimoto

**Affiliations:** 1 Space Sciences Laboratory, University of California Berkeley, Berkeley, CA USA; 2 Planetary Plasma and Atmospheric Research Center, Tohoku University, Sendai, Japan; 3 Lockheed Martin Solar and Astrophysics Laboratory, Palo Alto, CA USA; 4 The Catholic University of America, Washington, DC 20064 USA; 5 National Institute of Information and Communications Technology, Koganei, Japan; 6 Graduate School of Science, Kyoto University, Kyoto, Japan

**Keywords:** Particle acceleration, Solar energetic particles, Turbulence, Coronal mass ejections, Interplanetary shocks

## Abstract

In gradual Solar Energetic Particle (SEP) events, shock waves driven by coronal mass ejections (CMEs) play a major role in accelerating particles, and the energetic particle flux enhances substantially when the shock front passes by the observer. Such enhancements are historically referred to as Energetic Storm Particle (ESP) events, but it remains unclear why ESP time profiles vary significantly from event to event. In some cases, energetic protons are not even clearly associated with shocks. Here, we report an unusual, short-duration proton event detected on 5 June 2011 in the compressed sheath region bounded by an interplanetary shock and the leading edge of the interplanetary CME (or ICME) that was driving the shock. While < 10 MeV protons were detected already at the shock front, the higher-energy (> 30 MeV) protons were detected about four hours after the shock arrival, apparently correlated with a turbulent magnetic cavity embedded in the ICME sheath region.
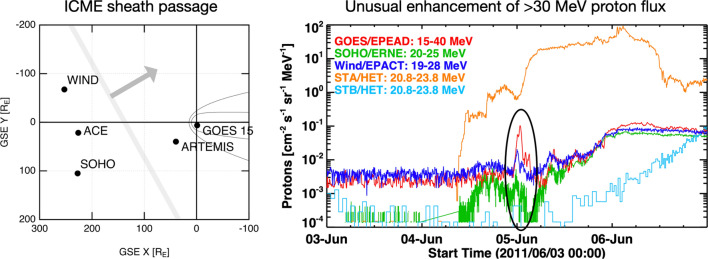

## Introduction

It is generally accepted that the shock waves driven by coronal mass ejections (CMEs) play a major role in accelerating particles in intense and space-weather-hazardous Solar Energetic Particle (SEP) events, which are often called “gradual” in the two-class scheme (e.g., Reames 1999, 2013; Desai and Giacalone 2016). After traveling the distance of several solar radii, the CME-driven shocks are commonly referred to as interplanetary (IP) shocks, which could also arise from stream-interaction regions. When an IP shock reaches near-Earth space, it not only signals a sudden storm commencement, but also is accompanied by an increase in energetic particle fluxes, as known since the 1960s (Bryant et al. 1962; Sarris and van Allen 1974; Gosling et al. 1981; Decker 1983; Scholer et al. 1983; Armstrong et al. 1985; Tsurutani and Lin 1985; Kennel et al. 1986; Reames 1999). Such particle events are collectively called energetic storm particle (ESP) events because of their temporal correlations with geomagnetic storms. However, ESPs are produced locally at the shock front and can be generally explained by the diffusive shock acceleration (DSA) theory in which the particle energy spectrum is determined by the shock strength (e.g., Giacalone 2012; Desai and Giacalone 2016).

ESP events may be classified into two types based on the intensity-time profiles, i.e., spike events and classic ESP events (Sarris and van Allen 1974), although additional varieties may exist including “step-like increase” and “irregular” events (Tsurutani and Lin 1985; Lario et al. 2003). Lario et al. (2003) studied ion data at 47–68 keV and 1–4.8 MeV, and electron data at 38–53 keV, and found that spike events more often appear in electron and lower-energy ion data than classic ESP events, consistent with the earlier findings by Sarris and Van Allen (1974). It is often thought that spike events are due to shock drift acceleration at the quasi-perpendicular shocks (e.g., Decker 1983).

However, energetic particles are not always associated with IP shocks (e.g., Lario et al. 2003; Reames 2012; Tessein et al. 2013). The association is particularly unclear for higher-energy (> 10 MeV) particles (Cohen et al. 2005). Instead, energetic particles can be associated with crossings of the heliospheric current sheet (HCS) (e.g., Richardson et al. 2006). Based on theories (Zank et al. 2014; Adhikari et al. 2019) and observations (Khabarova et al. 2015, 2016, 2017), it has been argued ﻿that recurrent magnetic reconnection occurs at the HCS and that particles are accelerated by dynamically evolving secondary current sheets and associated magnetic islands (or flux rope in 3D).

In this paper, we report on an unusual, short-duration enhancement of energetic protons that may at first look be classified as a spike-type ESP event based on its time profiles alone. A close inspection revealed, however, that it occurred in a magnetic cavity in the downstream of an IP shock. Our observations consist of energetic particle data from *Wind* (Acuña et al. 1995)*, Solar and Heliospheric Observatory* (SOHO) (Domingo et al. 1995) and *Geostationary Operational Environmental Satellite* (GOES) as well as the solar wind data from *Wind, Advanced Composition Explorer* (ACE; Stone et al. 1998), and *Acceleration, Reconnection, Turbulence, and Electrodynamics of the Moon’s Interaction with the Sun* (ARTEMIS; Angelopoulos 2011). We first describe the event in the context of the solar activity and near-Earth space environment, showing that it was unrelated to contemporaneous ‘gradual’ SEP events. We then describe the short-duration proton enhancement in detail and attempt at explaining how the protons were accelerated to high energies in the downstream region of the IP shock.

## Observation

**Fig. 1:**
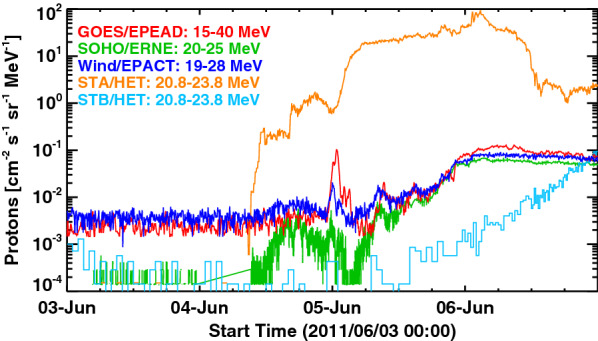
Overview of the energetic proton observations by GOES, SOHO, Wind, STEREO-A, and STEREO-B from 3 to 6 June 2011. The GOES satellite measured an unusual enhancement of protons in the 15–40 MeV energy range at around 0 UT on 5 June

The proton event in question was observed around 00 UT on 5 June 2011 at the first Lagrangian point (L1) and geosynchronous orbit (GEO). Before discussing the event itself, we describe how we isolated it from the ongoing SEP events. In Fig. 1, we show time profiles of proton flux at similar energies from detectors at five locations, including the twin spacecraft of *Solar Terrestrial Relations Observatory* (STEREO; Kaiser et al. 2008), i.e., STEREO-A (STA) and STEREO-B (STB), which were located at 95 degrees west and 93 degrees east of the Sun–Earth line. During 4–6 June 2011, the proton flux was much more elevated at STA than at L1 (or GEO) and STB due to two SEP events that were attributable to fast CMEs at 06:48 UT and 22:05 UT on 4 June. These SEP events have been already discussed by Lario et al. (2013) and Lawrence et al. (2014) with a particular focus on energetic electrons and neutrons, respectively, but we focus on energetic protons in this paper.

When measured by *Large Angle and Spectrometric Coronagraph Experiment* (LASCO) onboard SOHO, the two CMEs were as fast as 1440 km/s and 2524 km/s (projected on the plane of the sky), respectively, and associated with flares at N15W140 and N17W148. From the STA location, these flares were disk events. The 195 Å EUV data obtained by EUV Imager (EUVI) onboard STA indicate that these flares would have been X1 and X8 flares if they had been observed by GOES (Nitta et al. 2013). The estimated CME speeds and flare magnitudes indicate that the second event was more energetic, which also reflected in the proton flux at STA. At STB, from where the flare region was far behind the east limb, only the second SEP event was seen, starting late on 5 June (light blue curve in Fig. 1). Around the Sun–Earth line, both two SEP events were seen, although at much lower levels than at STA (red, green and blue curves). In particular, the *Energetic and Relativistic Nuclei and Electron* (ERNE) instrument onboard SOHO detected the two SEP events more clearly than *Electron, Proton, Alpha Detector* (EPEAD) onboard GOES because of its lower background level.

The spiky proton event we study was observed during the decay of the first SEP event and before the onset of the second SEP event at Earth around 03:30 UT on 5 June (see the profile of SOHO/ERNE). Note that it stands out most prominently in the GOES data. At L1, it is considerably weaker in the Wind data and hardly noticeable in SOHO/ERNE data although SOHO/ERNE has much lower background level. The *Solar Isotope Spectrometer* (SIS) onboard ACE also recorded the event in the 10–30 MeV and 30–80 MeV channels, but the data are available only as quick-look and browse data, so we do not include them in this study. We can rule out the possibility that the second SEP event mentioned above may have contributed to our event, because we expect the onset time (with respect to the CME on the Sun) and rise time to the peak to be correlated—there is no reason to expect a sharp increase of the particle flux long after the CME occurred. In this case, the proton rise time was only of the order of 10 min, whereas the onset time was delayed by more than two hours after the CME. Then, an eruption from a well-connected longitude may have produced a prompt event with short rise time like our event. However, there was no flare or CME around the time of the spiky proton event. Therefore, we expect this event to be produced in situ rather than escaping from an IP shock close to the Sun.

**Fig. 2:**
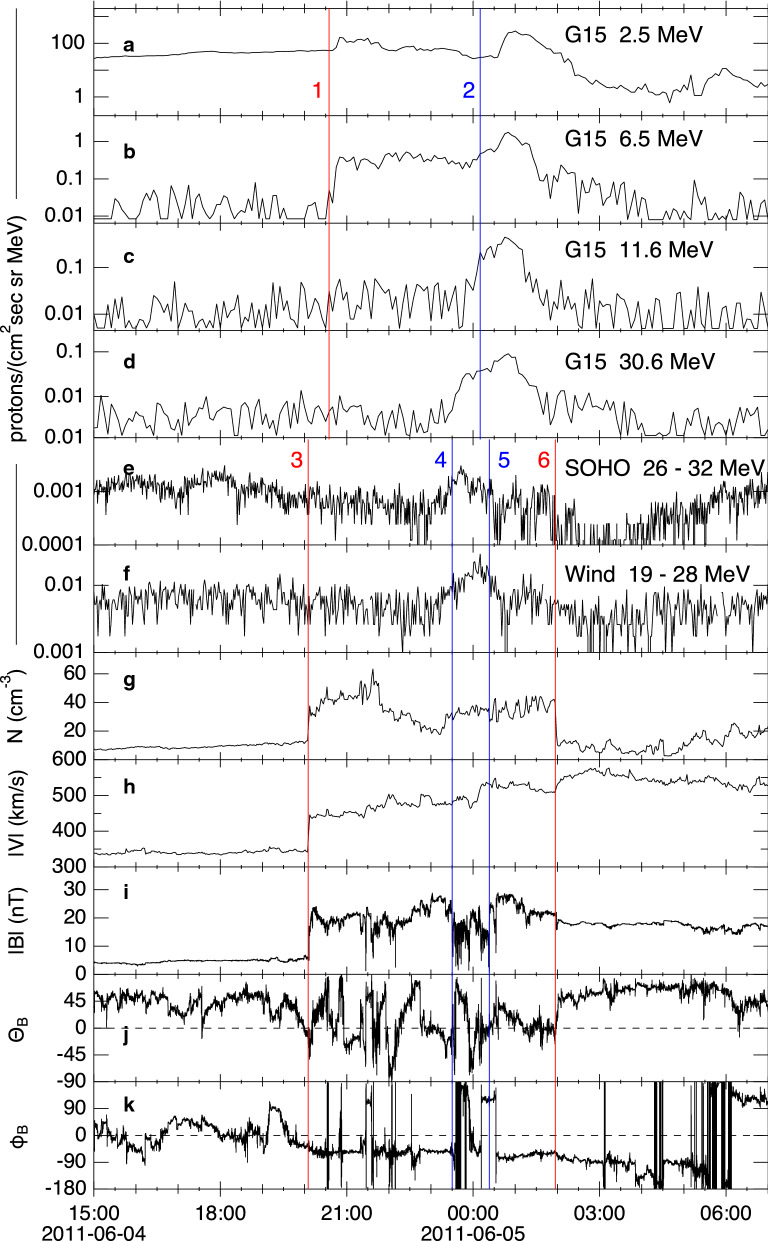
Enlarged view of the enhanced proton flux and associated plasma parameters. **a**–**d** Corrected proton flux measured by GOES at energies ~ 2.5, ~ 6.5, ~ 11.6, and ~ 30.6 MeV. **e**, **f** Energetic proton flux measured by SOHO in the 26–32 MeV range and by Wind in the 19–28 MeV range. **g**–**k** Various parameters of the solar wind measured by Wind. **g** Proton number density. **h** Proton flow speed. **i** Magnetic field magnitude (|*B*|). **j** Magnetic field latitudinal angle (*θ*_B_). **k** Magnetic field longitudinal angle (*φ*_B_). The data are shown in the Geocentric Solar Ecliptic (GSE) coordinate in which X-points toward the Sun, Z parallel to the ecliptic pole, and Y completing the right-hand system (toward dusk)

Now, we compare the temporal variations of energetic protons and solar wind parameters to see if our proton event was associated with an IP shock. Figure 2 plots proton fluxes at several energies and solar wind plasma and magnetic field from 15:00 UT on 4 June to 07:00 UT on 5 June. The top four panels (a)–(d) show the proton fluxes from GOES in four energy ranges from 2.5 to 30.6 MeV. The proton fluxes from SOHO in the 26–32 MeV range and Wind in the 19–28 MeV channel are plotted in panels (e) and (f). Panels (g)–(k) shows the solar wind parameters obtained by Wind.

We immediately spot the arrival of a shock wave around 20:06 UT on 4 June at the Wind satellite as indicated by the vertical line labeled 3. Yang et al. (2019), in a statistical study of electron acceleration at IP shocks detected by Wind, described this shock in detail, showing the enhancement of 0.1–4 MeV protons as well as 0.2–40 keV electrons across the shock front. This IP shock had quasi-perpendicular geometry, and the electron energy spectra were interpreted with shock drift acceleration (Yang et al. 2019). The shock was likely driven by the halo CME at 07:40 UT on 2 June 2011 that resulted from two successive eruptions at S19E25 involving NOAA active regions 11,226 and 11,227 (Zhang et al. 2015; Palmerio et al. 2017). The speed of the CME projected on the plane of the sky was 976 km/s as measured in SOHO/LASCO data. During its early evolution, this CME did not produce any SEP events at L1, STA or STB (Kihara et al. 2020).

As the IP shock propagated to the magnetosphere, the 6.5 MeV protons increased at GEO around 20:40 UT (Fig. 2b, line 1). This was coincident with the sudden storm commencement. The flux at this energy stayed high until 01:30 UT on 5 June, passing a second jump around 00:10 UT (line 2) which was more prominent at higher energies (Fig. 2c). However, soon after this second jump (approximately 1 h), the flux decreased rapidly. At higher energies (in particular ~ 30.6 MeV), the proton flux started to increase even before 00:10 UT. It is to be noted that GOES/EPEAD measures energetic proton flux in its eastward and westward directions and, in general, they can differ due to the geomagnetic cutoffs. However, in this event, the fluxes were comparable and showed very similar time profiles due likely to the enhanced solar wind dynamic pressure and subsequent inner motion of the geomagnetic cutoffs (Rodriguez et al. 2010). Thus, the average of the two fluxes is shown in Fig. 2.

A similar, short-duration enhancement was also seen at L1. The proton flux in the > 20 MeV range started to increase ~ 40 min earlier as marked by line 4 (Fig. 2e, f), and this ~ 40 min difference is comparable to our estimate of the time it took for the solar wind to propagate from L1 to Earth. Note that SOHO/ERNE detected other enhancements even before the shock arrival (owing to its lower background level) but they can be attributed to a separate SEP event, as described above.

The short-duration enhancement of energetic proton flux at L1 occurred in the compressed sheath region bounded by the IP shock that arrived about four hours earlier and an ICME flux rope that started around 02:00 UT on June 5 (line 6). The leading edge of this ICME appears to be a reverse-shock-like structure with sharp decreases of the density and magnetic field, correlated with a sharp increase of the flow speed [see Tsurutani et al. (2018) for another example of a “reverse wave” at the leading edge of an ICME]. Thus, the density was relatively high in the sheath region. Also, the solar wind speed increased monotonically across the sheath, indicating that this sheath region was, in fact, being compressed from behind and evolving.

During the time period of the energetic proton enhancement, the magnetic field magnitude of the solar wind dropped substantially and fluctuated rapidly (between lines 4 and 5 in Fig. 2). Because the temporal drop was correlated with the proton enhancement, the drop (which we hereafter refer to as magnetic cavity) will be examined in more detail later. Here, we note that there was a solar wind velocity jump at the peak time of the proton flux, i.e., 00:09 (at Wind) on 5 June, from |*V*|~ 480 to ~ 530 km/s. The density remained relatively steady across this jump, indicating that this velocity jump was not causing a substantial plasma compression.

**Fig. 3:**
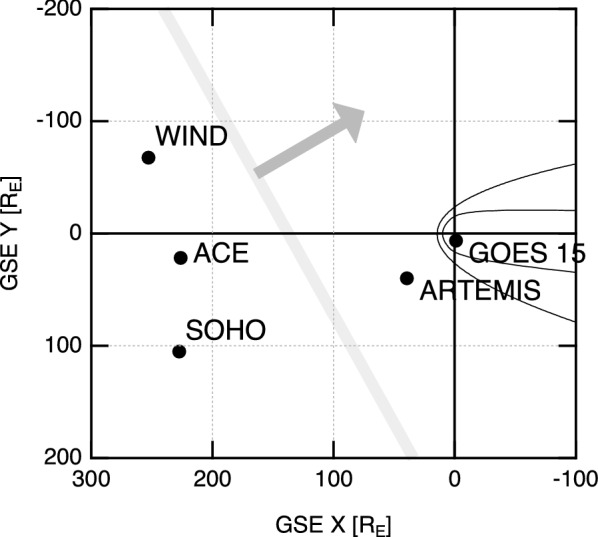
Spacecraft positions and estimated orientation of the interplanetary structure (thick gray line). The thin curves show the typical locations of Earth’s bow shock and magnetopause (Fairfield 1971)

To better understand the plasma environment associated with the short-duration, energetic proton enhancement, we studied the orientation of the observed structures using data from Wind, ACE, and ARTEMIS. Figure 3 shows the positions of the relevant spacecraft around the time of the passage of the velocity-jump structure. While GOES was in the magnetosphere, ARTEMIS was in the pristine solar wind and measured basically the same structure. Thus, we used data from Wind, ACE, and ARTEMIS and applied the two-dimensional timing method assuming that the structure was planar and the direction of motion was parallel to its normal direction. The derived normal direction was *n* ~ (− 0.89, − 0.46) and the propagation speed was *V* ~ 439 km/s. This result is also shown by the gray line and arrow in Fig. 3. Similar results were obtained when we used the timing of detection of the shock structure (at 6/4 20:06 at Wind in Fig. 2; *n* ~ (− 0.79, − 0.61), *V* ~ 432 km/s) and the reverse-shock-like structure (at 6/5 01:58 at Wind in Fig. 2; *n* ~ (− 0.87, − 0.49), *V* ~ 307 km/s). The estimated orientation is reasonable considering the fact that this IP shock has been attributed to solar eruption events at S19E25. While we interpreted earlier that the ICME sheath region was being compressed from behind and evolving, the substantially smaller speed of the reverse-shock-like structure further suggests that the ICME sheath region was, in fact, expanding and hence the reverse-shock-like structure was propagating sunward in the ICME-sheath rest frame.

Based on the 6-h difference between the forward shock and reverse-shock-like structure (i.e., between lines 3 and 6), the thickness of the compressed region (or ICME sheath) is estimated to be at least ~ 1040 *R*_E_, (= 307 km/s times 6 h) which is much larger than the gyroradius of ~ 30 MeV protons with the magnetic field magnitude of ~ 15 nT, i.e., ~ 8 *R*_E_. Also, the estimated propagation speeds are roughly equal to the instantaneous solar wind speed, indicating that the time profiles in Fig. 2 are spatial variations rather than temporal variations. In fact, the 40-min difference mentioned above (the difference between lines 1 and 3) as well as the peak time difference (the difference between lines 2 and 4) can be roughly explained by the travel distance between Wind and GOES.

**Fig. 4:**
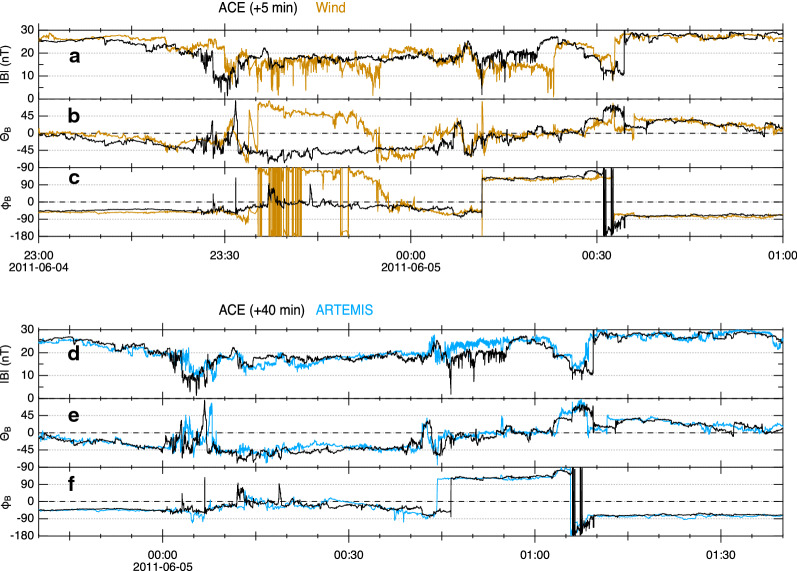
Enlarged view of the magnetic cavity embedded in the ICME sheath. **a**–**c** Variation of the magnitude (|*B*|), latitudinal direction (*θ*_B_), and longitudinal direction (*φ*_B_) of the magnetic field vector measured by ACE (black) and Wind (brown). **d**–**f** Same as **a**–**c** but measured by ACE (black) and ARTEMIS (blue). To account for the solar wind propagation, the ACE data are shifted in time by 5 and 40 min for **a**–**c** and **d**–**f**, respectively. In d and e, data from P1 (THB) of the twin spacecraft mission ARTEMIS are shown with the time resolution of ~ 0.25 s. Due to artificial noise in the *φ*_B_ component of this data product, data from P2 (THC) are used in f with the time resolution of ~ 3 s

Let us now examine more details of the magnetic cavity identified above. Figure 4 shows zoomed-in views of the magnetic cavity observed by Wind (brown curves), ARTEMIS (blue curves), and ACE (black curves), demonstrating a few notable features of the magnetic fluctuations in the cavity. Here, the ACE data are shifted in time to account for the solar wind propagation between spacecraft. It is evident that all spacecraft observed various fluctuations in the cavity. For example, at 00:12 UT, Wind and ACE detected a current-sheet-like structure, i.e., a sharp drop in the magnetic field magnitude (Fig. 4a) and a large rotation of the magnetic field direction (Fig. 4c). The same magnetic shear was also observed by ARTEMIS at 00:44 UT (Fig. 4e, f). However, discrepancies can also be found between ACE and Wind (Panels a, b, and c). For example, in the Wind data, there was a magnetic field rotation during 23:35–23:55 on 4 June but not in the ACE data. While the latitudinal angle (*θ*_B_) varied from ~ 80° to ~ − 60°, the longitudinal angle (*ϕ*_B_) varied from ~ 180° to ~ − 30°, although the two angles remained steady in the middle 23:40–23:50. There were also much shorter-scale (a few second) variations throughout the cavity in the Wind data (but much less frequently in the ACE and ARTEMIS data). Many of these shorter-scale variations are the so-called magnetic holes in which magnetic field magnitude decreases down to a few nT (e.g., Turner et al. 1977; Winterhalter et al. 1994; Haynes et al. 2015; Roytershteyn et al. 2015; Volwerk et al. 2020; Madanian et al. 2020). Magnetic holes are typically characterized as localized (a few second duration) depressions in the magnetic field magnitude with little (< 10°) rotation. It has been reported that magnetic holes are often observed in a high-beta condition associated with stream-interaction regions.

**Fig. 5:**
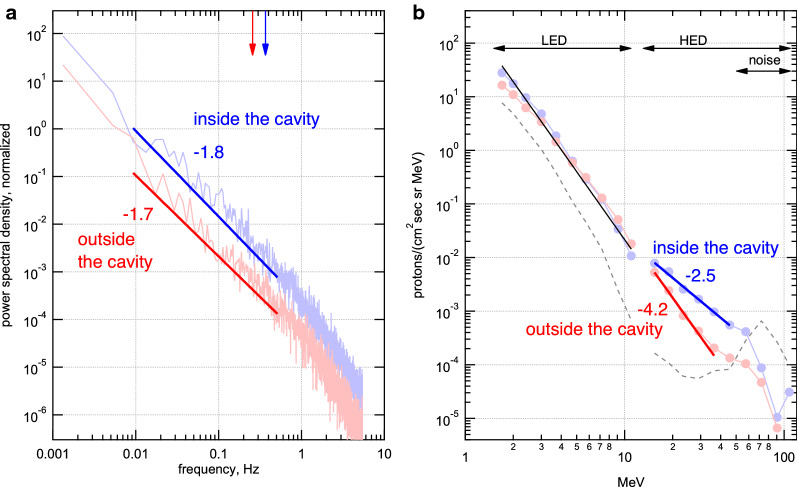
Magnetic field frequency spectra (left) and proton energy spectra (right) before (red) and during (blue) the observation of the magnetic cavity, providing additional perspective of the proton spike event. The frequency spectra are obtained from the GSE-z component of the magnetic field measured by the Wind spacecraft during 20:27:30–22:54:00 UT (outside the cavity; pink), and 23:20:30–24:33:00 (inside the cavity; light blue) of 4 June 2011. The ion cyclotron frequencies are indicated with the arrows. The energy spectra are obtained by the Low and High Energy Detectors of SOHO/ERNE during 22:00–23:00 UT (outside the cavity; red) and 22:00–23:00 UT (inside the cavity; blue). The background spectrum is obtained from a pre-SEP time period, i.e., 19:00–22:00 UT of 3 June 2011 (dashed curve) and is subtracted from the shown spectra. In both panels, both the power-law fit and spectral index are shown for each spectrum. The two proton energy spectra are almost identical below 10 MeV and have the power-law index of − 4.2 (black line)

Because of the presence of the various magnetic fluctuations during the proton flux enhancement, we further examined the frequency spectrum and hence the turbulence properties. Figure 5a shows the magnetic field spectra obtained just before (pink; 4 June 2011 22:30:00–23:15:00 UT) and during (light blue; from 4 June 2011 23:30:30 to 5 June 2011 00:30:00 UT) the observation of the magnetic cavity, demonstrating the presence of developed turbulence. Here, the power spectral densities are normalized by the background magnetic field magnitude squared. It is evident that the spectra show a power law and that the turbulence was enhanced in the cavity. In the frequency range near and below the ion cyclotron frequency (the arrows), the power law indices before (red) and during (blue) the cavity were ~ 1.7 and ~ 1.8, respectively, comparable to the typical value in the solar wind.

Figure 5b shows the background-subtracted, proton energy spectrum obtained by SOHO/ERNE, demonstrating that the spiky flux enhancement above 10 MeV (i.e., inside the cavity, colored blue) exhibited a power-law spectrum that is harder than that of before the spike (i.e., outside the cavity, colored red). Also shown for reference is the background spectrum (dashed curve) which contained a bump in the highest energy range (> 50 MeV) due to an artificial noise. The spectrum in the < 10 MeV range stayed almost the same across the cavity, suggesting that the acceleration mechanism in the cavity was different from the mechanism that produced < 10 MeV protons. Another caveat is that the SOHO/ENRE was also detecting a decay phase of an unrelated SEP event, as described earlier and shown in Fig. 1. Thus, to minimize the effect of the SEP, we used a narrow time period to show the pre-spike spectrum, i.e., 22:00–23:00 UT of 4 June 2011, as opposed to the entire time period of ICME sheath region before the spike.

## Summary and discussion

To summarize, we observed an unusual, short-duration proton event at both L1 and GEO, with a spectrum extending to energies beyond 30 MeV. This event was isolated from the contemporaneous SEP events and related to the passage of the shock wave from a CME that was launched a few days earlier. Also, the energetic particles appear to be trapped in the turbulent magnetic cavity in the ICME sheath region downstream of the shock. Therefore, we conclude that this spiky proton event was due to local acceleration at the magnetic structure embedded in the ICME sheath region and is distinct from those classically referred to as ESP events.

To examine how unusual this event was, we visually inspected GOES proton flux since 1999 and found several other cases of short-duration enhancement in the > 10 MeV range. However, all of them were more closely associated with IP shocks rather than a magnetic cavity as was the case on 5 June 2011. Therefore, an enhancement of ~ 30 MeV proton flux in an ICME sheath region is a very unusual phenomenon at 1 AU.

The precise mechanism of acceleration remains unclear, but we speculate that the lower-energy protons produced at the shock front served as seed particles and were further accelerated stochastically in the magnetic cavity in the shock downstream region. This is because particles were already accelerated up to ~ 6.5 MeV at the shock front and the magnetic cavity contained enhanced turbulence that may scatter and confine those particles (Fig. 5a). We also confirmed that the proton flux exhibited a power-law energy spectrum in SOHO/ERNE data (Fig. 5b). Because a stochastic process is necessary to explain a power-law spectrum (Fermi 1949), this turbulence embedded in the magnetic cavity may have played a role in confinement and subsequent acceleration of particles (e.g., Zank et al. 2014).

A closer inspection of the turbulence in the cavity revealed that there were different types of magnetic fluctuations in the cavity ranging from the large-scale (but smaller than ICME) magnetic field rotations (> 100 *R*_E_) to the small-scale (but larger than the kinetic scale) magnetic holes (~ 1000 km). Because these scales are too large or too small when compared to the gyroradius of 6.5 MeV protons, i.e., ~ 4 *R*_E_ for 15 nT, it is unlikely that these structures directly produced 30 MeV protons through wave-particle interaction or resonance. Nevertheless, recent particle simulations demonstrated that small-scale magnetic holes can be produced out of decaying turbulence (Roytershteyn et al. 2015). Thus, the above-mentioned detection of the magnetic holes in the 5 June event may be an indication that, prior to the observation, the turbulence level in the cavity was higher than that shown in Fig. 5a. Thus, even though relatively large fluctuations existed outside the cavity observed at around 21:20–22:20 UT of 4 June (and the power spectrum averaged over the entire sheath region before the cavity was comparable to that of the cavity; not shown), the cavity may have been more favorable for acceleration protons.

Here, we also note that the fluctuations of different scales were found at the Wind location (*y* ~ -67 *R*_E_) and not at the ACE (y ~ 22 *R*_E_) and ARTEMIS (y ~ 40 *R*_E_) locations. Therefore, it is likely that the cavity region was not spatially uniform and contained a sub-region of enhanced turbulence that were observed by Wind but not by ACE and ARTEMIS.

A puzzle remains as to what key process led to the enhancement of turbulence and/or what alternative mechanism accelerated protons up to 30 MeV if the turbulence alone was not able to accelerate protons. The velocity jump that coincided with the flux peak at Wind reminds us of the diffusive shock acceleration (DSA) theory whereby particles receive net energy gain through repeated collisions with waves in both upstream and downstream of a shock front (e.g., Blandford and Eichler 1987 and references therein). However, we consider that this velocity jump did not achieve a DSA-like mechanism. In DSA, the energy gain of a particle ∆*E*_up_ is achieved through head-on collisions with the waves moving toward the shock in the upstream region while the energy loss ∆*E*_dn_ occurs through head–tail collision with the waves moving away from the shock. The net energy gain is achieved because the upstream bulk flow is faster than the downstream bulk flow and thus |∆*E*_up_| >|∆*E*_dn_|. However, in our case of the velocity jump at 00:00 UT of 5 June, the propagation speed was comparable to the solar wind speed so that, when we properly shift to the jump-rest frame, the upstream speed (estimated to be ~ 13 km/s) is actually smaller than the downstream speed (estimated to be ~ 37 km/s). Thus, this velocity jump would have worked against acceleration. This interpretation is consistent with the fact that the density remained relatively steady during this period (Fig. 2g).

Another key process may be magnetic reconnection which is a fundamental plasma process that converts magnetic energy into particle energies. During 23:40–23:50 UT across which Wind observed a magnetic field rotation, the orientation of the magnetic field at Wind (i.e., *θ*_B_ ~ 45°, *ϕ*_B_ ~ 170°) was almost opposite to that at ACE (i.e., *θ*_B_ ~ − 45°, *ϕ*_B_ ~ 0°), suggesting that there was a current sheet between Wind and ACE. This current sheet may had been dynamically evolving via reconnection because it was immediately followed by fast solar wind around 00:00 UT of 5 June, as described above. In fact, it has been shown that magnetic reconnection and associated flux ropes (or magnetic islands in 2D) could be a key mechanism of the recently reported, Atypical Energetic Particle Events (AEPEs) (e.g., Khabarova et al. 2015, 2016, 2017; Adhikari et al. 2019). In AEPEs, energetic particles of up to several MeV are observed with no clear association with shock fronts but in the vicinity of the heliospheric current sheet (HCS). An HCS can exhibit complex structures after interacting with corotating interaction regions (CIRs) and/or ICMEs. Such complexity may lead to magnetic cavity, enhanced turbulence, and particle acceleration (Khabarova et al. 2017). Similarly, the event of 5 June was found in an ICME sheath region with a reverse-shock-like structure at the leading edge of the ICME. Therefore, the ICME sheath was also a CIR region. As such, this event may be categorized as an AEPE. However, we emphasize that the event of 5 June was unique in that the proton energies reached > 30 MeV, i.e., much larger than the energies of protons in previously reported AEPEs and ESP events, and that it was clearly associated with a magnetic cavity with turbulence.

To fully understand the enhancement of > 30 MeV protons in this event, it is necessary to examine more detailed properties of the observed turbulence. Also, it would be helpful to perform numerical modeling of the event using magnetohydro-dynamic (MHD) simulations combined with test particle simulations. Such tasks are well beyond the scope of this paper and left for future work.

## Data Availability

The GOES 15 particle data are produced by the NOAA Space Weather Prediction Center (SWPC) and are distributed by the NOAA National Geophysical Data Center (NGDC). The SOHO/ERNE data was made available by Space Research Laboratory at University of Turku, Finland. All other data were retrieved from NASA Coordinated Data Analysis Web (CDAWeb). For the Wind/EPACT data, key parameters were used upon consultation with D. Reames.

## Abbreviations

SEP: Solar Energetic Particle
CME: Coronal mass ejection
ESP: Energetic storm particle
AU: Astronomical unit
L1: The first Lagrangian point
GEO: Geosynchronous orbit
DSA: Diffusive shock acceleration
MHD: Magnetohydro-dynamics
